# A trait‐based approach to plant species selection to increase functionality of farmland vegetative strips

**DOI:** 10.1002/ece3.5047

**Published:** 2019-04-01

**Authors:** Claire J. Cresswell, Heidi M. Cunningham, Andy Wilcox, Nicola P. Randall

**Affiliations:** ^1^ University Centre Sparsholt Winchester, Hampshire UK; ^2^ Corteva Agriscience™ Abingdon, Oxfordshire UK; ^3^ Harper Adams University Newport, Shropshire UK

**Keywords:** biological control, ecosystem services, environmental factors, field margin, plant characteristics, plant traits, pollination, water quality protection

## Abstract

1. Farmland vegetative strips are a proven source of support for ecosystem services and are globally used to mitigate effects of agricultural intensification. However, increasing pressures on agricultural land require increases in their functionality, such as supporting multiple ecosystem services concurrently.
2. The plant species sown in a vegetative strip seed mix determine the establishment, plant community, and ecosystem services that are supported. Currently, there is no clearly defined or structured method to select plant species for multifunctional vegetative strips.
3. Plant traits determine how plants support ecosystem services. Also, the establishment and persistence of plant communities is influenced by key internal and external factors. We propose a novel, evidence‐informed method of multifunctional vegetative strip design based on these essential traits and factors.
4. This study had three distinct stages. The first identified plant traits that support water quality protection, pollinators and/or crop pest natural enemies, using existing research evidence. We then identified key factors affecting plant community establishment and persistence. Finally, we applied these standardized methods to design a multifunctional vegetative strip for a specific case study (UK lowland farmland).
5. Key plant traits identified, included floral display size, flower color, nectar content, leaf surface area, leaf trichome density, percentage fine roots, root length, rooting depth, and root density. Key internal and external establishment factors included life history, native status, distribution, established competitive strategy, associated floristic diversity, flowering time and duration, and preferred soil type and pH. In the United Kingdom case study, we used five different plant traits and all of the identified factors to design a seed mix for a multifunctional vegetative strip.
6. We present a transferable method of vegetative strip design that can be adapted for other ecosystem services and climates. It provides landowners and advisors with an evidence‐informed approach to increase field margin functionality while supporting farmland biodiversity.

**OPEN RESEARCH BADGES:**

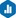

This article has earned an Open Data Badge for making publicly available the digitally‐shareable data necessary to reproduce the reported results. The data is available at https://doi.org/10.5061/dryad.8t52n38.

## 1 INTRODUCTION

Agricultural land use covers 37.4% of global land area as of 2015 (FAO, 2018). Farming it effectively for food production is vital for a globally expanding human population (Godfray et al., 2010; UN Population Division, 2018). Recent research has shown that achieving efficient agricultural production requires regulating ecosystem services, including pollination and biological control (biocontrol), which support the provisioning service of food production (Aizen, Garibaldi, Cunningham, & Klein, 2009; Blitzer, Gibbs, Park, & Danforth, 2016; Zavaleta, Pasari, Hulvey, & Tilman, 2010). However, wildlife declines have led to a reduction in the support for these services (Biesmeijer et al., 2006; Brown & Paxton, 2009; Carvell, Meek, Pywell, & Nowakowski, 2004; Dabrowski, Peall, Reinecke, Liess, & Schulz Runoff, 2002; Davies, 2000; Garratt et al., 2013; Gevao, Semple, & Jones, 2000; Kremen, Williams, & Thorp, 2002; Rusch et al., 2016; Stanley, Gunning, & Stout, 2013; Williams & Osborne, 2009; Winfree, Aguilar, Vázquez, LeBuhn, & Aizen, 2009). An example of this are declines in both pollinator abundance and diversity, and the plants that support them, which have led to pollination deficits in crops such as oil‐seed rape, watermelon, and apple (Biesmeijer et al., 2006; Brown & Paxton, 2009; Carvell et al., 2004; Garratt et al., 2013; Kremen et al., 2002; Stanley et al., 2013; Williams & Osborne, 2009; Winfree et al., 2009). Simplified, intensive agricultural landscapes have also been shown to have reduced natural enemy abundances, leading to a 46% lower level of crop pest control (Rusch et al., 2016). In addition, since 1945 increased applications have led to pesticides, together with nitrates, phosphates and sediment, polluting farmland water quality through runoff, erosion, and leaching to groundwater (Dabrowski et al., 2002; Davies, 2000; Gevao et al., 2000). This is of particular importance in the United Kingdom as just 35% of rivers are classified as “Good” according as of 2016 (Priestley & Barton, 2018).

To support ecosystem services and protect wildlife, while meeting food production requirements, a “sustainable intensification” approach has been proposed (Firbank, Elliott, Drake, Cao, & Gooday, 2013; Wentworth, 2008). This involves increasing food production from the existing agricultural land while minimizing pressure on the environment (Garnett & Godfray, 2012). One mechanism of this would be to increase the functionality of off‐crop habitats, such as vegetative strips in field margins, that support valuable ecosystem services within the farm, including water quality protection, pollination, and biocontrol (Haaland, Naisbit, & Bersier, 2011; Lye, Park, Osborne, Holland, & Goulson, 2009; Pfiffner & Wyss, 2004; Reichenberger, Bach, Skitschak, & Frede, 2007). Wildflower vegetative strips can increase pollinator visits to the crop by 25% (Feltham, Park, Minderman, & Goulson, 2015). If sown with grasses and wildflowers, they can provide shelter and food resources for natural enemies, which can reduce pest‐induced crop damage and increase yield to adjacent crops (Gurr, Wratten, & Barbosa, 2010; Tschumi et al., 2016). Also, vegetative strips sown along farmland watercourses are a proven method of water quality protection (Davies, 1999; Dorioz, Wang, Poulenard, & Trevisan, 2006; Haukos, Johnson, Smith, & McMurry, 2016; Muscutt, Harris, Bailey, & Davies, 1993; Reichenberger et al., 2007). As a result, farmers in Europe are required to buffer any waterbody next to arable land with a 2m wide vegetative strip under the Common Agricultural Policy and Water Framework Directive (DEFRA, 2014; European Commission, 2018). They often have very low botanical diversity (Mayer, Reynolds, McCutchen, & Canfield, 2007), but studies have shown that the introduction of other plant species should not affect water quality protection (Cole, Brocklehurst, Robertson, Harrison, & McCracken, 2015; Critchley, Fowbert, Sherwood, & Pywell, 2006; Mayer et al., 2007). The current available evidence in literature does not demonstrate diversity of plant species as a key factor in the provision of ecosystem services, but rather the individual plant species and their morphological traits (de Bello et al., 2010; Díaz et al., 2007; Kattge et al., 2011; Lavorel & Garnier, 2002; Lavorel et al., 2013; Violle & Jiang, 2009; Violle et al., 2007). Mayer et al. (2007) discovered that buffer strips of various vegetation types, including forest, forested wetland, herbaceous, herbaceous/forest, and wetland, were equally effective at removing nitrogen from soils. Consequently, there is scope to sustainably increase the number of ecosystem services that vegetative strips support while still provisioning for wildlife. This could aid food production in the face of mounting restrictions on land availability and pressures on landowners and wildlife (Hackett & Lawrence, 2014; Stutter, Chardon, & Kronvang, 2012).

Some attempts at integrating support for different ecosystem services have been made (e.g., Biddinger & Rajotte, 2015), but the potential to provide water quality protection and support for pollinators and natural enemies in one vegetative strip has been little explored. The plant species included in a vegetative strip seed mix will determine the establishment, resulting plant community and therefore ecosystem services that are provided. From current literature, there is no evidence of a clearly defined or structured method of plant species selection for vegetative strips. Numerous seed companies, charities, and other organisations provide seed mix options and advice to support biodiversity or ecosystem services (e.g., Syngenta, 2014; Buglife, 2018; Kings Seeds, 2018; Emorsgate Seeds, 2018). Typically, these were developed by observation and experience in the field (Nowakowski & Pywell, 2016), but this method is not transparent, structured, or repeatable. Evidence‐informed decision support tools have been developed for general farming practices (e.g., Centre for Ecology & Hydrology, 2018), but so far, none exist for selecting plant species for multifunctional vegetative strips.

Plant functional traits and their uses in determining species performance, in predicting changes in community compositions and their effect on ecosystem functioning, are increasingly being investigated (de Bello et al., 2010; Díaz et al., 2007; Lavorel & Garnier, 2002; Lavorel et al., 2013; Violle & Jiang, 2009; Violle et al., 2007). The specific morphological traits of a plant, or effect traits as defined by Lavorel and Garnier (2002), such as nectar content, floral display size, or leaf area (Kattge et al., 2011), determine how it supports specific ecosystem services (Díaz et al., 2007; Garnier & Navas, 2012). For example, Bianchi and Wackers (2008) showed that more parasitoids were attracted to plants with a higher nectar content, Kudo, Ishii, Hirabayashi, and Ida (2007) showed that a larger floral display size was preferred by *Bombus hypocrita *supsp. *Sapproensis *and Burylo, Dutoit, and Rey (2014) showed that a plant's leaf area positively correlated with its ability to trap sediment. In addition, internal factors, such as the life history of a plant species, and external factors, such as the established competitive strategy of plant species in the same community, can significantly affect the establishment of the desired plant community. For example, if a plant species has a perennial life history it should return each year (Marshall & Moonen, 2002), and if noncompetitive grasses are sown with the forbs, this could enhance the chance of the desired forbs establishing (Laskey & Wakefield, 1978). Therefore, they should also be considered when selecting species for a seed mix.

There are many sources of plant trait and internal/external factor data for UK species, (e.g., Fitter & Peat, 1994; Grime, Hodgson, & Hunt, 2007; Baude et al., 2016; Biological Records Centre, 2018), providing an extensive evidence base for plant species selection. There are also reviewing methods, such as systematic mapping, that provide a structured and comprehensive process to discover evidence that may explain which specific plant traits support the target ecosystem services.

In the pursuance of designing a vegetative strip to support multiple ecosystem services, we propose a novel, evidence‐informed method which utilizes plant traits and key establishment factors, which can be applied to a wide range of farmland environments within temperate climates. The target ecosystem services to be supported by this vegetative strip include water quality protection, pollination, and biocontrol.

## 2 MATERIALS AND METHODS

This study was undertaken in three distinct stages. The first stage identified plant traits that support water quality protection, pollinators, and/or crop pest natural enemies, using existing research evidence. The second stage identified internal (concerning the plant itself) and external (concerning the environment) factors essential for plant community establishment and persistence within a vegetative strip. Stage three applied the standardized methods from the first and second stages to a specific case study for lowland farmland within the United Kingdom, where plant species were selected for a multifunctional vegetative strip.

### 2.1 Stage One: The identification of plant traits that support the target ecosystem services

We used a standardized, systematic reviewing method to collate existing research on plant traits that support the target ecosystem services. A systematic map approach was used as it is a transparent, repeatable, structured, and unbiased method to collate evidence (Collaboration for Environmental Evidence, 2018; Grant & Booth, 2009). The exact methods used to carry out the systematic map can be found in Blowers, Cunningham, Wilcox, and Randall (2017).

In summary, a combination of published peer‐reviewed and gray (i.e., noncommercially available) literature sources were comprehensively searched using specific key terms to capture an unbiased sample of the literature. Articles were considered relevant where they investigated a plant trait and its provision of the target ecosystem services in a temperate region. Any experimental or correlative study, that collected primary data and that met the above criteria, was included in the database (Cresswell, Cunningham, Wilcox, & Randall, 2018).

Each article was categorized using a combination of generic (e.g., country of study, publication date, authors) and topic specific (e.g., plant trait, target organism, and ecosystem service provided) keywords. Only findings from studies that met predefined critical appraisal requirements (i.e., adequate replication or randomisation of samples and no clear confounder), were used to inform the final assessment of the plant traits. For each included study, the specific plant trait, target organism and outcome were identified. Data were extracted from the map to make cross‐comparisons between the findings to build a robust evidence base for plant species selection.

### 2.2 Stage Two: Identification internal and external factors that aid in establishment and persistence of plant communities

The establishment and persistence of plant communities is influenced by key internal and external factors (Grime et al., 2007; Laskey & Wakefield, 1978). Internal factors could include preferred soil type or the plant's competitive nature. External factors could include the soil type in which the seed mix is sown or the associated floristic diversity of other establishing species in the vegetative strip. Plant morphological traits define how a plant species may support specific ecosystem services (Díaz et al., 2007; Garnier & Navas, 2012). However, establishment of the desired plant species will determine the support provided by a vegetative strip. Therefore, the key influencing internal and external factors must be identified and considered during plant species selection.

A group of topic experts in factors affecting establishment and persistence of plant communities were consulted to investigate what specific data should be gathered in order to include them in the plant selection process. Information sources were searched, including Laskey and Wakefield (1978), Landis, Wratten, and Gurr (2000), Marshall and Moonen (2002), Grime et al. (2007), Wentworth (2008), Kirk and Howes (2012) and Biological Records Centre (2018). Data extracted from these sources were collated and used to develop a table of initial criterion that each plant species should pass through before they are considered for inclusion in a seed mix.

### 2.3 Stage Three: Case Study on UK plant species

Information from stages one and two were applied to a case study, in this case UK lowland farmland. We compiled a list of all UK, native, perennial forbs, and grasses that showed an indication of good distribution across the United Kingdom, according to the Online Atlas of the British and Irish Flora (Biological Records Centre, 2018). Data on their traits (identified in stage one) and internal and external factors affecting establishment (identified in stage two) were then collected and coded into a database. The full database and details on the sources searched for this information can be found in Supporting information Appendix S1.

Internal and external factors affecting establishment identified in stage two formed an initial criterion for plant species selection. Plant species were then ranked relative to their ability to aid in the provision of the target ecosystem services (water quality protection, and support for pollinators and natural enemies) according to the traits already identified. Some of the factors from stage two were weighted for importance in lowland temperate environments. The ranks for each plant species were totaled and those with the highest rank carried forward to be considered for inclusion within a final multifunctional seed mix. The plant communities were developed so that a range of plant traits would be present.

## 3 RESULTS

### 3.1 Stage One: Overview of the systematic map

**Figure 1:**
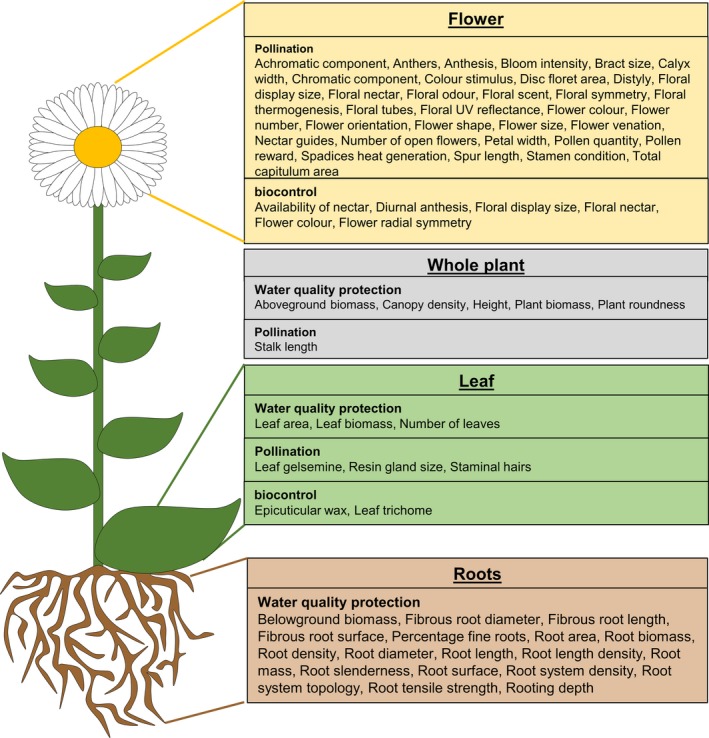
Plant traits and related ecosystem services investigated in the literature. (Data adapted from Cresswell et al. (2018)

From a total of 11,705 from the initial search, 56 articles met all the relevant criteria to be included for data extraction. Data extracted from the systematic map report (Cresswell et al., 2018), on the identified plant traits and their corresponding ecosystem service, are shown in Figure 1.

Pollinator support was the most commonly studied ecosystem service and many of the included articles investigated plant traits that focussed on different aspects of the floral display of a plant, for example, floral display size (*n* = 11), flower color (*n* = 9), and flower shape (*n* = 3). Some of the articles collated on crop pest natural enemy support also studied flower color and floral nectar. Both floral and leaf traits such as flower radial symmetry and leaf shape were found to influence invertebrates. Out of the articles collated on water quality protection, 17 related to the roots and root system of the plant.

Articles that studied the same plant trait all drew the same conclusions, for example, the articles investigating floral display size all identified that a larger display was preferred by the test species of pollinator (Table 1).

**Table 1:** Data extracted from the systematic map showing the important aspects for the chosen plant traits and the corresponding references. The full references can be found in Supporting information Appendix S2

Plant trait	Aspect of trait	Target organism/system	Outcome	Reference
Floral display size	Larger	*Apis mellifera*, Bombus sp., Osmia sp., Bombylius sp., Usia bicolor, Diptera, Hymenoptera, Coleoptera, Heteroptera, Lepidoptera, Syrphidae, Pollinators, Flower visiting insects	Preference shown	Shykoff & Bucheli (1995); Galen (1996); Johnson & Dafni (1998); Møller & Sorci (1998); Elle & Carney (2003); Sánchez‐Lafuente & Parra (2009); Barrio & Teixido (2015)
	Larger	Flying hawkmoth	Increased reproduction of plant	Herrera (1993)
	Larger	*Bombus hypocrita* subsp. *Sapproensis*	Increased attractiveness	Kudo et al. (2007)
	Larger	Pollinators	Attracted more	Ohashi & Yahara (2004)
	Larger	Andrena spp., *Anthophora acervorum, Apis mellifera, Bombus impatiens, Bombus pascuorum, Bombus pratorum, Bombus terrestris*, Pollinators, Muscid and Anthomyiid flies, Syrphidae, Others	Increased visitation	Conner & Rush (1996); Totland (2004); Sánchez‐Lafuente et al. (2005); Brunet et al. (2015); Garbuzov & Ratnieks (2015)
Flower color	Yellow	Crab spiders, Coleoptera, Syrphid flies (Allograpta and Platycheirus)	Preference shown	Campbell et al. (2010); Rocha‐Filho & Rinaldi (2011); Reverte et al. (2016)
	UV‐yellow	Ants, wasps & diptera	Preference shown	Reverte et al. (2016)
	White	Crab spiders, Solitary bees (Hylaeus), Coleoptera, Pollinators	Preference shown	Campbell et al. (2010); Mu et al. (2011); Rocha‐Filho & Rinaldi (2011); Reverte et al. (2016)
	Blue	*Philoliche aethiopica*	Preference shown	Jersáková et al. (2012)
	Pink	*Usia bicolor*, Crab spiders, Lepidoptera	Preference shown	Johnson & Dafni (1998); Rocha‐Filho & Rinaldi (2011); Reverte et al. (2016)
	Ultramarine blue/Bee‐UV‐blue	*Melipona mondury*	Preference shown	Koethe et al. (2016)
	Bee‐green	*Melipona quadrifasciata*	Preference shown	Koethe et al. (2016)
	Green	Ants	Preference shown	Reverte et al. (2016)
	Color change	*Bombus hypocrita* subsp. *Sapproensis*	Susceptible to display patterns and floral display size	Kudo et al. (2007)
	Purple	Bees	Preference shown	Reverte et al. (2016)
	Red	Pollinators	Preference shown	Shang et al. (2011)
Nectar content	Higher	*Aphidius ervi*, Bees and flies	Preference shown	Ashman et al. (2000); Vollhardt et al. (2010)
	Higher	*Apis mellifera, Andrena nigrihirta, Andrena tridens, Andrena carlini, Nomada perplexa, Xylocopa virginica virginica, Augochlora pura, Augochlorella striata*, *Osmia conjuncta, Osmia lignaria*, Dialictus sp., Osmia sp., Honeybees, Bumblebees, Parasitoids	Attracted more	Motten (1983); Bianchi and Wackers (2008); Schmidt et al. (2015)
Leaf area	Larger	Soil erosion	Reduced soil erosion	Burylo et al. (2012b)
	Larger	Sediment	Reduced soil erosion	Burylo et al. (2014)
	Larger	Runoff, soil erosion, sediment & sediment concentration	Reduced soil erosion	Chau & Chu (2017)
	Larger	Rainfall interception	Increased	Li et al. (2016)
	Larger	N & P removal	Increased N & P removal from soil	Read et al. (2010)
Leaf trichomes	More	Pea leaf weevils	Increased abundance	Chang et al. (2004)
Percentage fine roots	Higher	Soil erosion	Reduced soil erosion	Burylo et al. (2012a)
Root length	Longer	Soil aggregate stability	Increased	Gould et al. (2016)
	Longer	Nitrate uptake	Increased nitrate uptake rate	Sullivan et al. (2000)
Rooting depth	Deeper	N & P removal	Increased N & P removal from soil	Read et al. (2010)
	Deeper	Nitrate uptake	Increase nitrate uptake rate	Sullivan et al. (2000)
Root density	Higher	Runoff, soil erosion, sediment & sediment concentration	Reduced soil erosion	Chau & Chu (2017)

### 3.2 Stage Two: Identified internal and external factors affecting plant community establishment and persistence

Information gathered on internal and external factors that were identified to affect the establishment and persistence of a multifunctional vegetative strip is shown in Table 2. Life history, Status, and Distribution formed the initial criterion that a plant species would be required to pass before being considered for inclusion in the seed mix.

**Table 2:** Internal and external factors affecting establishment, their desirable aspect for a multifunctional vegetative strip, the justification and the associated reference. Factors highlighted in bold determined whether a plant species could be considered for inclusion within the seed mix

Factor	Aspect	Justification	Reference
Life history	Perennial	Vegetative strips along farmland watercourses should last 5–10 years, without resowing, so annuals are not suitable	Marshall and Moonen (2002)
Status	Native	To avoid introduction of invasive non‐natives	Wentworth (2008)
Distribution	Regional	Well‐regionally distributed will ensure seed is more widely applicable within the region	Biological Records Centre (2018)
Established competitive strategy	Noncompetitive	Grasses have been shown to outcompete wildflowers, so their competitive strategy must be considered	Laskey and Wakefield (1978)
Associated floristic diversity	High	High associated floristic diversity increases the chance of wildflowers establishing well	Grime et al. (2007)
Flowering time and duration	Duration of beneficial invertebrate season of activity	To provide pollen and nectar sources throughout season	Landis et al. (2000)
Soil type	Suitable for varied types	To ensure growth and good establishment of the plant	Grime et al. (2007); John Szczur, GWCT
Soil pH	Suitable for varied soil pH	To ensure growth and good establishment of the plant	Grime et al. (2007), John Szczur, GWCT
Suitability to native beneficial invertebrates	High	To ensure selected species provide support for the target beneficial invertebrates	For example, Kirk and Howes (2012)

### 3.3 Stage three: United Kingdom plant species case study, the ranking system and the results of the application

The traits and remaining establishment factors used to rank each plant species are detailed in Table 3.

**Table 3:** Plant trait and factor ranking and weighting system used to identify suitable forbs and grasses for a multifunctional seed mix

	Plant trait/factor	Ranking parameter and suitability value	Data source
Forbs	Floral display size^a^	0: <10 mm, 1: ≥10 mm	Baude et al. (2016)
	Trichome density	0: Sparse, 1: Numerous	Grime et al. (2007)
	Leaf area	0: <25 mm^2^, 1: ≥25 mm^2^	Grime et al. (2007)
	Root system	0: Tap‐root, 1: Adventitious	Fitter and Peat (1994); Grime et al. (2007)
	Leaf phenology	0: estival, 1: Evergreen	Fitter and Peat (1994); Grime et al. (2007)
	Soil type	0: Not suitable for most soils, 5: Suitable for most soils. These scores were heavily weighted as suitability to most soil types was essential for establishment of the multifunctional vegetative strip in varying conditions.^b^	Expert advice: John Szczur, GWCT cross‐referenced with data from Grime et al. (2007); Biological Records Centre (2018)
Grasses	Leaf area class	1: <15, 2: 15–20, 3: 20–25, 4: 25–30, 5: >30 mm^2^	Grime et al. (2007)
	Established strategy	0: C or SC or CR, 1: CSR or R or S or SR Where C = Competitor, R = Ruderal, S = Stress‐tolerator, CR = Competitive‐Ruderal, SC = Stress‐tolerant Competitor, SR = Stress‐tolerant Ruderal and CSR = C‐S‐R strategist	Grime et al. (2007)
	Height (maximum)	0: ≥2,000, 1: 1,500–2,000, 2: 750–1,500, 3: ≤750 mm	Fitter and Peat (1994)
	Associated floristic diversity	1: 10.0 species or fewer, 2: 10.1–14.0, 3: 14.1–18.0, 4: 18.1–22.0, 5: >22.0	Grime et al. (2007)

a Size of total floral display, not individual florets

b This ranking parameter can be adapted to target specific soil types, for example, targeting a sandy loam soil—0: not suitable for sandy loam soil, 5: suitable for sandy loam soil.

Forbs ranked highly if they had a large floral display size and leaf surface area, leaves with numerous trichomes, an adventitious root system, and evergreen leaves. All grasses were required to have an adventitious root system but also scored highly if they had a large leaf surface area, a less competitive established strategy, a lower comparative height, and a high associated floristic diversity. Once the higher scoring forbs and grasses were identified they were then combined to create the final seed mix.

All plant species highlighted in Tables 4 and 5 were included in the seed mix for the multifunctional vegetative strip. In these tables, the heavy weighting of the factor “suitability to most soil types” was necessary as soil conditions can vary hugely from farm to farm. In addition, plants that can establish in a range of soil types, are more appropriate for a mix such as this, as a specific, designed plant community is desired. In addition, due to cost restrictions and standard practice, the seed mix consisted of 20% forbs and 80% grasses. An alternative mix was also created with a ratio of 50% forbs and 50% grasses to investigate the effect of this difference on establishment of the desired community. Two further multifunctional plant mixes were developed, one for a heavy clay soil and one for a sandy loam soil. The same method was used, with the exception that rankings took into account plant suitability for the respective soil types. This was to test whether designing a seed mix bespoke to a specific soil type would better encourage the establishment of the desired plant community.

**Table 4:** Grasses assessed for inclusion in the multifunctional seed mix and their corresponding ranks. Overall rank is also displayed as the sum total of the ranks for each plant trait/factor. All plant species highlighted in the table were included in the multifunctional vegetative strip seed mix

Botanical name	Leaf area	Established strategy	Height (maximum)	Associated floristic diversity	Overall rank
*Agrostis capillaris*	5	1	3	2	11
*Festuca pratensis*	4	1	2	4	11
*Phleum pratense*	5	0	1	3	9
*Dactylis glomerata*	4	0	2	3	9
*Alopecurus pratensis*	4	0	2	3	9
*Festuca rubra *agg.	2	1	2	3	8
*Festuca arundinacea*	2	0	0	4	6

**Table 5:** Forbs assessed for inclusion in the multifunctional seed mix and their corresponding ranks. Overall rank is also displayed as the sum total of the ranks for each plant trait/factor. All plant species highlighted in the table were included in the multifunctional vegetative strip seed mix. Ranked forbs all showed signs of support for all groups of bees according to Kirk and Howes (2012)

Botanical name	Floral display size	Trichome density	Leaf area	Root system	Leaf phenology	Soil type	Total
*Trifolium pratense*	1	1	1	0	1	5	9
*Trifolium repens*	1	0	1	1	1	5	9
*Centaurea nigra*	1	1	1	0	0	5	8
*Taraxacum officinale *agg.	1	0	1	0	1	5	8
*Stachys sylvatica*	1	1	1	0	0	5	8
*Leucanthemum vulgare*	1	0	0	1	1	5	8
*Prunella vulgaris*	0	1	1	?	1	5	8
*Lotus corniculatus*	1	0	1	0	0	5	7
*Daucus carota*	0	0	1	0	1	5	7
*Achillea millefolium*	0	0	1	0	1	5	7
*Galium verum*	0	1	0	?	1	5	7
*Ranunculus acris*	1	0	0	0	0	5	6
*Silene dioica*	1	0	0	0	0	5	6
*Veronica chamaedrys*	1	1	1	1	1	0	5
*Hypochaeris radicata*	1	1	0	1	1	0	4
*Primula vulgaris*	1	1	1	?	1	0	4
*Heracleum sphondylium*	1	1	0	0	0	0	2
*Vicia cracca*	0	1	1	0	0	0	2
*Potentilla erecta*	1	?	0	1	0	0	2
*Scrophularia nodosa*	?	0	1	0	0	0	1
*Knautia arvensis*	1	0	0	0	0	0	1
*Malva moschata*	?	0	0	0	1	0	1
*Potentilla anserina*	1	0	0	0	0	0	1
*Geranium pratense*	1	0	0	0	0	0	1

Note

“?” denotes where data were not available on the plant trait for a specific plant species.

## 4 DISCUSSION

The knowledge gaps identified by the systematic map emphasize a need for additional research to be undertaken in these areas. However, the articles that were included provided sufficient evidence to inform the plant species selection. In addition, the concurrence of the findings in the articles in the systematic map allowed increased confidence in the evidence used in the plant species selection process.

For some of the plant traits identified in Stage One, the information relating to their presence or absence in individual UK plant species was unavailable. For example, the research identified specific traits such as fibrous root length or depth as indicative of an adventitious root systems to aid water quality protection type, but only the overall root system could be identified (e.g., in Grime et al., 2007). This influenced what could be presented in the database of UK plant species (Supporting information Appendix S1). In other cases, the data available on traits were incomplete for some plant species (indicated by “?” in Table 5) potentially impacting an individual species ranking. For plant species where the trait information is lacking, further primary research, would strengthen this method of vegetative strip design. Screening experiments could be undertaken to record measurements of specific plant trait parameters such as maximum and minimum size of floral display.

Although the three‐stage approach identified the top scoring plants, other UK lowland farmland‐specific issues were also considered. The commercial availability of the seed affected the final seed viable mixes. Where this was an issue lower scoring plants that covered a similar flowering period were substituted. For example, two high scoring UK forbs, lady's bedstraw (*Galium verum*) and self‐heal (*Prunella vulgaris*), could not be sourced from seed companies and so were not included in the multifunctional seed mix, Table 5. A slightly lower scoring plant, primrose (*Primula vulgaris*), though not guaranteed to grow well in all soil types, was included because it has many of the desirable traits, but also flowers early in the season and some higher scoring plants do not. Similarly, the grass species Cock's foot (*Dactylis glomerata*), had a slightly lower score than some others due to its competitive nature, but was included as its pollen is often gathered by pollinators (Kirk & Howes, 2012).

The plant species chosen for these seed mixes were all selected for use within the United Kingdom; however, the methods used can be applied to other temperate regions by choosing plant species native to that country. The TRY Plant Trait Database created by Kattge et al. (2011) can be used to access information gathered from numerous plant trait databases across the world.

There is a common misconception that the diversity of a vegetative strip may increase its potential to support ecosystem services; however, Birkhofer et al. (2018) found a lack of general relationship between multifunctionality and diversity. Instead, this study focussed on developing functionality of vegetative strips through the use of what specifically defines a plant's ability to support ecosystem services, their traits (Kattge et al., 2011). If a mixture of forbs and grasses are sown instead of a grass only mix, and the factors influencing establishment of the desired community are controlled, then diversity will naturally increase none the less.

The method outlined in this study could be used to develop seed mixes that target other ecosystem services also. In particular, drought or flood tolerance, while not feasible in such a mix as this, the method could be applied to develop a seed mix that targets this specific service. Further, systematic mapping of plant traits that support drought or flood tolerance could be undertaken and the information used to inform the method of design outlined here.

With a seed mixture containing forbs and grasses, a management regime of cutting once in early summer and once in late would be recommended. This would aid the control of competitive grass species growth (Grime et al., 2007) and avoid removal of the floral resources during the peak season of pollinator and natural enemy activity (Kirk & Howes, 2012). In addition, removal of the cuttings after each cut is recommended, to reduce the nutrient load in the soil (Crofts & Jefferson, 2007). This would further encourage the forbs to establish as they require conditions of lower fertility (Grime et al., 2007).

## 5 CONCLUSIONS

In this study, we have outlined and demonstrated an evidence‐informed method to design multifunctional vegetative strips. By using this three‐stage approach for the first time in vegetative strip design, we have developed a method that focusses on exactly what is required of individual plants, and of plant communities, to support ecosystem services in farmland. This method is widely applicable to different environmental conditions within temperate farmland and allows a more informed decision‐making process when choosing plant species for vegetative strip seed mixes.

In‐field experiments are currently underway to test the long‐term establishment and viability of the test seed mixes. If establishment of the desirable plant community is achieved and sustained, then this method of vegetative strip design could be a proven, useful tool that could inform agricultural environmental policies. For example, the European Common Agricultural Policy does not currently stipulate that vegetative strips, along farmland watercourses, need to be sown with anything but a standard grass seed mix (European Commission, 2018). If payments to farmers could be offered as an incentive to sow a more enhanced, multifunctional seed mix along watercourses on their land, this could positively affect biodiversity within farmland while increasing support for regulating services to the farmer. Field margins need to become multifunctional due to restricted land availability, increased food production requirements, and farmland biodiversity declines. This novel method will allow landowners to increase the functionality of their field margins or other vegetative strips by supporting three vital ecosystem services, while re‐introducing biodiversity into the landscape. The method has the potential to be adapted for other ecosystem services and climate zones.

## CONFLICT OF INTEREST

The authors declare that they have no competing interests.

## AUTHOR'S CONTRIBUTIONS

CJC: Principle researcher who undertook the literature searches in stages one and two, development of plant species trait database and design and application of plant species ranking system in stage three and write‐up of manuscript. AW: General support of contributing project and review of draft manuscripts. HMC: General support of contributing project and review of draft manuscripts. NPR: Project concept and management. Guidance and support through all stages of the work. Manuscript design and editing.

## Data Availability

Data available from the Dryad Digital Repository.
