# Supplementary material for: A trait‐based approach to plant species selection to increase functionality of farmland vegetative strips
Source: Ecol Evol. 2019 Apr 1;9(8):4532–43. doi: 10.1002/ece3.5047 (PMC6476755; doi:10.1002/ece3.5047)
Supplement: Supplementary file 2 [file ECE3-9-4532-s002.docx]

**References for Table 1 - Data extracted from the systematic map showing the important aspects for the chosen plant traits.**

Ashman, T.L., Swetz, J., Shivitz, S., 2000. Understanding the basis of pollinator selectivity in sexually dimorphic Fragaria virginiana. Oikos. 90, 347-356.

Barrio, M., Teixido, AL., 2015. Sex-dependent selection on flower size in a large-flowered Mediterranean species: an experimental approach with Cistus ladanifer. Plant Syst. Evol. 301, 113-24.

Bianchi, F., Wackers, F.L., 2008. Effects of flower attractiveness and nectar availability in field margins on biological control by parasitoids. Biol. Control. 46, 400-408.

Brunet, J., Thairu, M.W., Henss, J.M., Link, R.I., Kluever, J.A., 2015. The Effects of Flower, Floral Display, and Reward Sizes on Bumblebee Foraging Behavior When Pollen Is the Reward and Plants Are Dichogamous. International Journal of Plant Sciences. 176, 811-819.

Burylo, M., Rey, F., Mathys, N., Dutoit, T., 2012a. Plant root traits affecting the resistance of soils to concentrated flow erosion. Earth Surf. Proc. Land. 37, 1463-1470.

Burylo, M., Rey, F., Bochet, E., Dutoit, T., 2012b. Plant functional traits and species ability for sediment retention during concentrated flow erosion. Plant Soil. 353, 135-144.

Burylo, M., Dutoit, T., Rey, F., 2014. Species Traits as Practical Tools for Ecological Restoration of Marly Eroded Lands. Restoration Ecol. 22, 633-640.

Campbell, D.R., Bischoff, M., Lord, J.M. and Robertson, A.W., 2010. Flower colour influences insect visitation in alpine New Zealand. Ecology. 91, 2638-2649.

Chang, G.C., Rutledge, C.E., Biggam, R.C., Eigenbrode, S.D., 2004. Arthropod diversity in peas with normal or reduced waxy bloom. J. Insect Sci. 4, 18.

Chau, N.L., Chu, L.M., 2017. Fern cover and the importance of plant traits in reducing erosion on steep soil slopes. Catena. 151, 98-106.

Conner, J., Rush, S., 1996. Effects of flower size and number on pollinator visitation to wild radish, Raphanus raphanistrum. Oecologia. 105, 509.

Elle, E., and Carney, R. 2003. Reproductive assurance varies with flower size in Collinsia parviflora (Scrophulariaceae). 90, 888-896.

Galen, C., 1996. Rates of Floral Evolution: Adaptation to Bumblebee Pollination in an Alpine Wildflower, Polemonium viscosum. Evolution. 50, 120-125.

Garbuzov, M., Ratnieks, F.L.W., 2015. Using the British National Collection of Asters to Compare the Attractiveness of 228 Varieties to Flower-Visiting Insects. Environ Entomol. 44 638-46.

Gould, I.J., Quinton, J.N., Weigelt, A., De Deyn, G.B., Bardgett, R.D., 2016. Plant diversity and root traits benefit physical properties key to soil function in grasslands. Ecology Letters. 19, 1140-9.

Herrera, C.M., 1993. Selection on floral morphology and environmental determinants of fecundity in a hawk moth‐pollinated violet. Ecol. Monogr. 63, 251-275.

Jersáková, J., Jürgens, A., Šmilauer, P., Johnson, S.D., Johnson, M., 2012. The evolution of floral mimicry: identifying traits that visually attract pollinators. Funct. Ecol. 26, 1381.

Johnson, S.D., Dafni, A., 1998. Response of bee‐flies to the shape and pattern of model flowers: implications for floral evolution in a Mediterranean herb. Funct. Ecol. 12, 289-297.

Koethe, S., Bossems, J., Dyer, A.G., Lunau, K., 2016. Colour is more than hue: preferences for compiled colour traits in the stingless bees Melipona mondury and M. quadrifasciata. Journal of Comparative Physiology A: Neuroethology, Sensory, Neural, and Behavioral Physiology. 202, 615-27.

Kudo, G., Ishii, H.S., Hirabayashi, Y., Ida, T.Y., 2007. A test of the effect of floral color change on pollination effectiveness using artificial inflorescences visited by bumblebees. Oecologia. 154, 119-128.

Li, X., Xiao, Q., Niu, J., Dymond, S., van Doorn, N.S., Yu, X., et al., 2016. Process-based rainfall interception by small trees in Northern China: The effect of rainfall traits and crown structure characteristics. Agricultural & Forest Meteorology. 218, 65-73.

Møller, A.P., Sorci, G., 1998. Insect preference for symmetrical artificial flowers. Oecologia. 114, 37-42.

Motten, A.F., 1983. Reproduction of Erythronium umbilicatum (Liliaceae): pollination success and pollinator effectiveness. Oecologia. 59, 351-359.

Mu, J., Li, G., Niklas, K.J., Sun, S., 2011. Difference in floral traits, pollination, and reproductive success between white and blue flowers of Gentiana leucomelaena (Gentianaceae) in an alpine meadow. Arct. Antarc. Alp. Res. 43, 410-416.

Ohashi, K.A.Z.U.H.A.R.U., Yahara, T.E.T.S.U.K.A.Z.U., 2001. Behavioural responses of pollinators to variation in floral display size and their influences on the evolution of floral traits. Cognitive ecology of pollination. Cambridge University Press, Cambridge. 274-296.

Read, J., Fletcher, T.D., Wevill, T., Deletic, A., 2010. Plant Traits that Enhance Pollutant Removal from Stormwater in Biofiltration Systems. Int. J. Phytoremediation. 12, 34-53.

Reverte, S., Retana, J., Gomez, J.M., Bosch, J., 2016. Pollinators show flower colour preferences but flowers with similar colours do not attract similar pollinators. Annals of Botany. 118, 249-57.

Rocha-Filho, L.C., Rinaldi, I.M.P., 2011. Crab spiders (Araneae: Thomisidae) in flowering plants in a Brazilian" Cerrado" ecosystem. Braz. J. Biol. 71, 359-364.

Sánchez-Lafuente, A.M., Guitian, J., Medrano, M., Herrera, C.M., Rey, P.J., Cerda, X., 2005. Plant traits, environmental factors, and pollinator visitation in winter-flowering Helleborus foetidus (Ranunculaceae). Annal. Bot. 96, 845-852.

Sánchez-Lafuente, A.M., Parra, R., 2009. Implications of a long-term, pollinator-mediated selection on floral traits in a generalist herb. Annal. Bot. 104, 689-701.

Schmidt, K., Filep, R., Orosz-Kovacs, Z., Farkas, A., 2015. Patterns of nectar and pollen presentation influence the attractiveness of four raspberry and blackberry cultivars to pollinators. Journal of Horticultural Science & Biotechnology. 90, 47-56.

Shang, Y., Venail, J., Mackay, S., Bailey, P.C., Schwinn, K.E., Jameson, P.E., Martin, C.R., Davies, K.M., 2011. The molecular basis for venation patterning of pigmentation and its effect on pollinator attraction in flowers of Antirrhinum. New Phytol. 189, 602-615.

Shykoff, J.A., Bucheli, E., 1995. Pollinator visitation patterns, floral rewards and the probability of transmission of Microbotryum violaceum, a venereal disease of plants. J. Ecol. 83, 189-198.

Sullivan, W.M., Jiang, Z.C., Hull, R.J., 2000. Root morphology and its relationship with nitrate uptake in Kentucky bluegrass. Crop Sci. 40, 765-772.

Totland, O., 2004. No evidence for a role of pollinator discrimination in causing selection on flower size through female reproduction. Oikos. 106, 558-564.

Vollhardt, I.M.G., Bianchi, F.J.J.A., Wäckers, F.L., Thies, C., Tscharntke, T., 2010. Nectar vs. honeydew feeding by aphid parasitoids: does it pay to have a discriminating palate? Entomol. Exp. Appl. 137, 1-10.
